# Robotic pilot study for analysing spasticity: clinical data versus healthy controls

**DOI:** 10.1186/s12984-015-0103-8

**Published:** 2015-12-02

**Authors:** Nitin Seth, Denise Johnson, Graham W. Taylor, O. Brian Allen, Hussein A. Abdullah

**Affiliations:** University of Guelph, 50 Stone Road East, N1G 2W1, Guelph, ON Canada; Hamilton Health Sciences, Regional Rehabilitation Centre, 300 Wellington Rd North, L8L 0A4, Hamilton, ON Canada

**Keywords:** Spasticity, Assessment, Modified ashworth scale, Dynamic time warping

## Abstract

**Background:**

Spasticity is a motor disorder that causes significant disability and impairs function. There are no definitive parameters that assess spasticity and there is no universally accepted definition. Spasticity evaluation is important in determining stages of recovery. It can determine treatment effectiveness as well as how treatment should proceed. This paper presents a novel cross sectional robotic pilot study for the primary purpose of assessment. The system collects force and position data to quantify spasticity through similar motions of the Modified Ashworth Scale (MAS) assessment in the Sagittal plane. Validity of the system is determined based on its ability to measure velocity dependent resistance.

**Methods:**

Forty individuals with Acquired Brain Injury (ABI) and 45 healthy individuals participated in a robotic pilot study. A linear regression model was applied to determine the effect an ABI has on force data obtained through the robotic system in an effort to validate it. Parameters from the model were compared for both groups. Two techniques were performed in an attempt to classify between healthy and patients. Dynamic Time Warping (DTW) with k-nearest neighbour (KNN) classification is compared to a time-series algorithm using position and force data in a linear discriminant analysis (LDA).

**Results:**

The system is capable of detecting a velocity dependent resistance (*p*<0.05). Differences were found between healthy individuals and those with MAS 0 who are considered to be healthy. DTW with KNN is shown to improve classification between healthy and patients by approximately 20 *%* compared to that of an LDA.

**Conclusions:**

Quantitative methods of spasticity evaluation demonstrate that differences can be observed between healthy individuals and those with MAS of 0 who are often clinically considered to be healthy. Exploiting the time-series nature of the collected data demonstrates that position and force together are an accurate predictor of patient health.

## Introduction

Upper motor neural (UMN) syndrome is common in those with multiple sclerosis, spinal cord injury, stroke, or other forms of acquired brain injury (ABI). It can manifest itself in the form of negative features, such as flaccidity and weakness, or positive features such as exaggerated tendon reflexes or spasticity [1]. Although spasticity has inconsistent definitions in literature [2], it can be described as a “sustained involuntary activation of muscles” and is commonly attributed to an exaggerated stretch reflex during passive stretch [3]. This abnormality of muscle tone becomes clinically apparent as spinal shock or ABI resolves and the individual begins to regain volitional control [4]. The presence of this abnormal tone is problematic for several reasons. First, it produces difficulties for the individual recovering as it inhibits their volitional abilities. Second, it is problematic for individuals trying to provide the patient with care for hygiene as well as rehabilitation. This abnormal tone can also lead to secondary complications such as muscle and soft tissue contracture and clonus [4, 5].

The most commonly accepted method to assess spasticity in individuals with UMN is the Modified Ashworth Scale (MAS) shown in Table 1 [4, 6]. Although widely used, the MAS has very poor sensitivity to changes in spasticity [7]. Many studies have assessed the validity and reliability of the Ashworth Scale and the MAS, and compared them to biomechanical measures [8–11]. These studies, while highlighting limitations, do suggest that both scales are appropriate for the upper limb (UL) spasticity evaluation, specifically the elbow joint. It is suggested, however, that these scales are still unable to represent small changes in spasticity necessary for treatment in clinical settings and are not an adequate gauge of the velocity dependent relationship [10]. Furthermore, patients with MAS scores of 0, which represents no tone, will still report issues associated with moving their limb. Thus, a more practical, descriptive, and quantitative measure of UL spasticity evaluation is desired. Quantitative information in particular should be able to reflect more subtle changes associated with an individual’s muscle tone and spasticity compared to a ranked categorical scale. Such information would provide a better description of an individual’s condition and how it is changing. This information will allow healthcare professionals to make more informed decisions regarding treatment options and monitor progress through an individual’s rehabilitation.

**Table 1:** The Modified Ashworth Scale

Score	Description
0	No increase in muscle tone
1	Slight increase in muscle tone, manifested by a catch and release or by minimal resistance at the end of the range of motion when the affected
	part(s) is moved in flexion or extension
1+	Slight increase in muscle tone, manifested by a catch, followed by minimal resistance throughout the remainder (less than half) of the ROM
2	More marked increase in muscle tone through most of the ROM, but affected part(s) easily moved
3	Considerable increase in muscle tone, passive movement difficult
4	Affected part(s) rigid in flexion or extension

### Previous work

One common school of thought is that the best way to monitor spasticity is using surface EMG readings to quantify neural activity in the affected limbs. This approach can be considered a logical first choice as the EMG can read the involuntary muscle activity that characterizes spasticity. In addition to measuring signals from EMG, it is also quite common for studies to complement these readings by additional biomechanical measures or techniques.

One of the earliest studies to use EMG to characterize spasticity was performed by Powers et al. [12] while employing a servo-controlled torque motor to manipulate the forearm with the shoulder flexed and abducted. This study tested the slope of torque against joint angles during passive limb extension as a measurement of joint-stiffness. It was concluded that stretch reflex threshold measures should be the basis of hypertonia assessment rather than measures from mechanical stiffness [12]. This idea of surface EMG-based assessment has also been supported in studies that compared healthy individuals to stroke patients [7, 13]. In particular, EMG studies have been performed for the UL to assess the prevalence of motor unit firing saturation [13]. It was found that differences between the EMG signals from healthy individuals and patients could be determined whereas differences in their force-profiles could not.

Limitations associated with EMG studies, however, are often noted. In particular, a very limited range of motion is often used in these studies which only provides insight and monitoring for the small range studied. Furthermore, there exist some well-known issues associated with surface EMG readings themselves including but not limited to environmental noise, placement issues, and changes in surface conduction due to sweat [14–16]. In addition, EMG readings are not a desirable tool to be used by frontline workers due to the additional need for wiring and setup time.

### Biomechanical focused studies

To improve upon initial findings with EMG-focused studies, new approaches have incorporated biomechanical measurements, usually forces or torques, to complement EMG readings. These approaches are usually performed with customized devices or motorized systems [7, 12, 17, 18]. Many strong contributions have been made using a combination of both EMG and biomechanical measures. One such method is described in Pisano et al. [18] who used a torque motor and sensor to evaluate spasticity as a slope of best fit versus position data. One strength of this study was that it was a follow-up clinical trial on patients that was compared to a previous study performed on a healthy population. The study also recorded neurophysiological measures and found several of them to be higher in the patient computation. Compared to the neurophysiological measures from EMG, however, the authors suggest that biomechanical measures had more promise at describing spasticity although the two could be used together.

Another approach that combined biomechanical measurements and EMG was demonstrated by Pandyan et al. [17]. who tested a spasticity quantification device comprising a force transducer and a flexible electrogoniometer to detect resistance in elbow flexion/extension. The elbow joint was selected because it was deemed a more reliable joint for the MAS evaluation [17]. In addition, the authors state that the MAS was not good for measuring spasticity but good for measuring resistance to passive movement. The 2003 follow up study aimed to address the clinical validity of the MAS based on 100 measures taken from 63 people [19]. A method of least squares best fit was performed to estimate the change in force against the change in elbow angle. Resistance to passive movement and velocity showed significant differences between the MAS 0 and MAS scores greater than zero. No significant differences were observed between MAS 1, 1+ and 2’s. Thus, it was demonstrated that it is possible to distinguish MAS 0’s using biomechanical measurements, but not the remaining groups of interest.

A similar study and technique was discussed in Malhotra et al. [7], also using a force transducer and flexible electrogoniometer but focused on the wrist. In addition, EMG readings were taken of long wrist flexors and extensors, and motions were performed at 2 distinct but uncontrolled velocities. Muscle activity was quantified by calculating the area under the curve of muscle activity versus passive range of motion. In this study, however, no clear pattern between muscle activation patterns and resistance were found [7].

### Proposed work

Several of the previous studies discussed are based on surface EMG readings. Limitations associated with studies employing surface EMG readings include performing movements in a limited range of motion, approximating the resistance as only a scalar slope, or not controlling the joint motions or their velocities. To address these limitations, we present a robotic system customized by physiotherapists (PTs) to quantify UL spasticity for elbow flexion/extension in the sagittal plane. As opposed to monitoring forces in a single plane, the robotic pilot study collects quantifiable position and force data in 3-dimensional Cartesian coordinates. This multidimensional approach is taken for two main reasons. First, it allows for methods of analysing direction of forces more carefully through its individual components as opposed to representing resistance as a single scalar value such as a slope. Secondly, multidimensional data is collected in an effort to detect cases when tone has changed but cannot be reflected in the MAS scale. This addresses situations where resistance is still present but manifesting in a different direction compared to previous assessments. Employing the approach of collecting data from a 3-dimensional Cartesian space may present unique profiles from forces exerted in these multiple directions. By considering all three dimensions it is expected that we can construct a more robust representation of force data related to spasticity.

This novel system is based on an adapted industrial robotic arm adapted for the primary purpose of spasticity assessment. This robotic system is different from others presented in several ways. First, it is focused on performing spasticity assessments in the sagittal plane for the upper limb. Clinicians identified upper extremity tone of the elbow flexors and extensors as key muscle groups that impact both fine and gross motor function. Additionally, these muscle groups are commonly targeted by clinicians for ongoing assessment and treatment. In addition, such a system also allows for customizable and controlled ranges of motion in the Sagittal plane. Another advantage of the system is the presence of the robotic arm’s internal position sensors capable of logging 3-dimensional data. These sensors provides a reduction in system complexity while the multidimensional data provides the opportunity to track a more descriptive representation of patient data. Finding more descriptive and sensitive methods to better describe spasticity is important as recovery time is very slow for those with an ABI. Treatment methods, such as Botox, Baclofen and surgery, and their respective evaluations require time which increases healthcare costs. An improved spasticity measure which is highly sensitive to change could allow physicians to make more accurate rapid decisions which would shorten each patient’s length of stay thereby reducing cost of care.

Results of this study provide evidence that the system is capable of distinguishing between healthy individuals and individuals with spasticity using this multidimensional data. Special emphasis is placed on differentiating between healthy individuals and individuals with MAS scores of 0 who normally would have been considered to have no abnormal tone. In addition, it is demonstrated that the accuracy of differentiating between healthy individuals and patients can be further improved by considering the multi-dimensional and temporal nature of the data collected as opposed to just averaged force readings alone.

In an effort to make the system more appealing to physiotherapists (PTs) and clinicians, system complexity and setup time was reduced by limiting the number of required sensors. This aspect makes the system easier to use in physiotherapy clinics and hospital environments. Furthermore, the exclusion of EMG signals avoids recognized acquisition difficulties including environmental noise, electrode conductivity changes and sensor location shifts common with surface electrodes [14–16]. This approach is an important departure from several methods outlined that rely on surface EMG readings to quantify or verify their results. Although needle-point EMG readings have been found to be accurate and present fewer issues compared to surface EMG readings, the extra procedure to insert the EMG increases the time and costs associated with such systems. In an attempt to develop a system that will actively be used by clinicians on the front line delivering rehabilitation, only biomechanical data alone is obtained to provide a more compatible set-up that will gain acceptability if relationships are found. EMG also requires additional staff training and resources and many clinicians that measure tone do not have EMG in their scope of practice. This would limit the adoption of a system that requires specialists to operate it. Our system can be used by a variety of health care providers and potentially even support staff such as assistants. As previously stated, systems also using EMG readings were not found to demonstrate the ability to differentiate amongst the different MAS scores in the middle of the scale or determine relationships with force.

This paper further advocates for using directional force and position data to improve the ability to distinguish between healthy and patient groups. Specifically, the temporal nature of the data collected lends itself to time series classification methods. Lastly, this paper attempts to determine the construct validity of the system through its ability to measure velocity dependent resistance.

## Methods

For this study, a Fanuc 6 Degree of Freedom (DOF) robotic arm was employed to manipulate the individual’s limb through passive elbow flexion/extension in the sagittal plane [20]. The main outcome measures collected by the robot were the time, position, and force data. The position and time readings were determined from the robotic arm’s internal position sensors and computer’s internal clock. Force readings were measured from a 6DOF force/torque sensor integrated into the system.

**Fig. 1:**
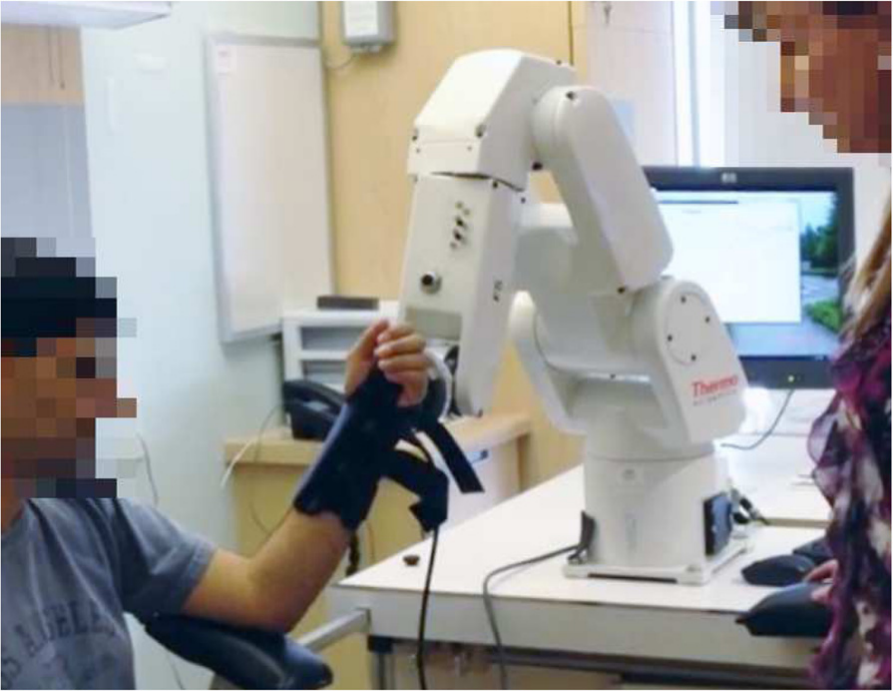
Arm configuration with robot arm and clinician. Individual is seated beside the robotic arm such that their flexion/extension motions pass by in front of the manipulator to cover their range of motion. A clinician is present with the patient at all times making eye contact while having access to emergency stops

The main outcome measures collected by the clinician were the MAS assessments performed in the moments prior to robot data collection. The individual was seated, usually in their specialized wheelchair for both MAS assessment and robot data collection. The use of personal specialized wheelchairs helped ensure that the trunk was stationary during the procedure. All individuals not in specialized wheelchairs were capable of trunk control and did not require constraints but were positioned in a supportive chair with a solid backrest and armrest. The individual’s elbow joint was placed on an adjustable armrest and was attached to the robot through a glove-like brace similar to those already in used by the rehabilitation centre. An example of this setup and configuration is shown in Fig. 1 showing both patient and clinician positioned with respect to the robot. For comfort, a bearing was placed in the end-effector to allow the individual small amounts of rotation between their limb and the system. During set up, the therapist moved the individual’s arm through their available range of motion in order to set the start and end points for the robotic movement. Once the desired flexion and extension positions were established, a path was approximated using an ellipse equation with the individual’s arm geometry forming the major and minor axes. These axes of different lengths allow for a comfortable flexion/extension motion.

Although the MAS is designed to be done in a supine position, this rarely occurs due the additional time and personnel required to place individuals in this position and then move them back into their custom wheelchair. This intervention has been designed to be easily and quickly adjustable to a seated position, which is currently typical practise and allows the patients to remain in their own custom wheelchair.

### Inclusion criteria

Forty-five healthy individuals, ages 28–65, with no UL impairments were recruited through word-of-mouth to participate in a healthy baseline data study. A second group of 40 individuals were recruited for a clinical trial. The participants for the clinical study were recruited from an in-patient acquired brain injury rehabilitation program at a regional tertiary care hospital in the province of Ontario, Canada. Individuals meeting the study criteria were identified by their treating physiotherapist and approached to participate in the study. Criteria were: i) radiological evidence of ABI; ii) between 18–65 years old; iii) MAS score between 0–3.

Participants were between 10 weeks and 2 years post injury and had confirmed radiological evidence of a brain injury. Eighteen of those participants were traumatic injuries and 22 were hemorrhagic injuries. There were 16 females and 24 males in the study. Of the 40 individuals, 33 had both limbs tested and 7 had only unilateral testing.

Prior to data collection, a PT not associated with the study collected informed consent from the participant or their guardian. All participants for the healthy study were in good active condition and did not have any ongoing medical issues. This study was approved by the Hamilton Integrated Research Ethics Board (REB) and the University of Guelph REB.

### Exclusion criteria

People with MAS score of 4 were excluded from the study as the amount of tone they possessed would immediately trigger the system’s safety stops. Individuals with acute orthopaedic injuries, or cognitive/behavioural issues that would prevent safe use of the robot were also excluded from this study. Individuals who experienced pain while moving their limb were also excluded.

### Data collection and system operation

From the 40 individuals, we collected a total of 88 limb recordings as several participants were able to have data collected from both limbs or were able to participate more than one time. Each limb was analysed as a separate entry with its own MAS scores and associated forces while still grouped according to the individual. Assessments were performed by a physiotherapist prior to collecting data with the system.

The system employs controlled passive elbow flexion/extension within each participants’ available range of motion as set by the PT. To remain consistent with the Lance definition of velocity dependent motions, the robotic arm passively leads the limb through 5 flexion/extension motions at increasing speeds. To ensure safety and comfort, 2 second pauses are introduced between each UL flexion and extension motion. Additional comments from the clinician can also be inputted if required.

The instantaneous speed measurements for each individual depended on their arm geometry as well as the amount of resistance they were exhibiting through the motion. On average, the final repetition was performed at a velocity of 20 cm/s 95 *%* CI (17.90,20.13) for the faster set of speeds. The final repetition, on average, for the slower set of speeds was performed at 15 cm/s 95 *%* CI (13.31,16.42). Using the robot’s controller, repetitions preceding the final motions were performed at 20,40,60 and 80 *%* of the individual’s maximum speed.

Forces and positions in each of the 3 Cartesian coordinates were measured from the tip of the robot arm where the individual was attached. These values were recorded every 15 ms with corresponding timestamps. Instantaneous velocity was then approximated from these values. All data collected was written to a database which was index by patient ID number, date and time of session. To ensure comfort, safety, and adequate data collection without triggering the safety stops, the set of speeds used was determined by the individual’s level of impairment and the discretion of the clinician. All healthy individuals performed their motions at the faster speed set.

### Average force comparison between healthy individuals and MAS 0

A Welch’s t-test was performed to determine whether a difference in average force existed between healthy individuals and patients with MAS scores of 0 as a result of their abnormal tone. The ability to determine differences and actively classify between these two groups at the end of a session is useful to PT’s and clinicians who often perform an assessment based on their abilities to manipulate an individual’s limb. Welch’s t-test was used due to the unequal variances within the two groups [21].

### Equality of speed effect slopes

To determine whether the velocity affected the average force of each individual, and whether this effect was different between the two groups, a linear regression model was constructed as shown in Eq. 1. Force *F* was regressed onto 7 explanatory variables. The variable *h*_*i*_ (*i*=1,2..6) represents the effect of an individual from the healthy clinical group or one of the first 5 levels of the MAS as listed in Table 1. *p*_*ij*_ (*j*=1,2,..,*n*_*i*_) represents the random effect of *j*th participant in the *i*th group. *l*_*ijk*_ indicates the right or left limb (*k*=1,2) for the *j*th participant in the *i*th group. *s*_*l*_(*l*=1,2,3) represents the effect of speed level discussed in System Operation section. *d*_*m*_ (*m*=1,2) represents the effect of the direction of motion (flexion or extension). *t*_*im*_ represents the effect of an interaction between *h* and *d*, and *r*_*im*_ represents the slope of the force that depends on group and direction, as the robot’s speed progressively increases (*X*=0.2,0.4,…,1.0).

(1) $$ F_{ijkl} = \mu + h_{i} + p_{ij} + l_{ijk} + s_{l} + d_{m} + t_{im} + r_{im}X + \epsilon_{ijkl}   $$

where *F*_*ijkl*_ is the average force magnitude of a single passive motion, *μ* is the average force of all participants, and *ε*_*ijkl*_ is the error associated with each observation with errors assumed to be uncorrelated and normally distributed with a mean of 0 and constant variance.

Each slope, *r*_*im*_, was tested for significance, with the null hypothesis *H*_0_ : *r*_*im*_=0 The slopes were also compared to determine if a difference exists between the slopes of healthy individuals compared to all patients as well as comparing healthy individuals to those with scores of MAS 0. An interaction between direction of motion and classification of an individual was also tested for the slopes.

The full model was fitted for MAS bicep scores and a second time with MAS tricep scores. Factors that were not found to have an effect on average force were removed one at a time to produce a reduced model for testing.

### Fisher’s linear discriminate analysis

One statistical method used to characterize two different classes from a dataset is Linear Discriminant Analysis (LDA). Using an appropriate selection of discriminators to represent each individual, an LDA can determine whether a linear combination of discriminators can separate the two groups as opposed to single values used in the t-tests.

Similar to an ANOVA, an LDA uses a within-class variance to determine a line **w** which can be used to discriminate between classes [22]. After calculating the means of a set based on discriminators of the entire dataset **m**, a new test case **x** can be classified to class 1 if **w**^*T*^(**x**−**m**)>0 and to class 2 otherwise.

This technique will also help determine whether a benefit exists in representing individuals as a collection of values based on multi-dimensional time series data as opposed to single values often provided by a 1-D sensor. Similar to a t-test, average force can be used again as a single discriminant to compare the two groups. This single discriminant method can then be compared to results from an LDA with multiple potential discriminators that are derived from the force, position and time data that can be used in combination to represent a single individual’s performance. A list of potential discriminators considered in this study are listed in Table 2.

**Table 2:** Discriminators used with Fisher’s LDA

Type (name)	Discriminators
Position Type (*X*_*maxForce*_)	X,Y,Z position in mm where max force occured
Mean Force ($\bar {F}$)	Mean Force reading in Newtons along X,Y,Z axes
	Mean Force Magnitude Value
Maximum Force (*F* *X*_*max*_)	Maximum force along X,Y,Z axes
	Maximum force magnitude
Force Slope ($\hat {F}$)	Slope of magnitude force across repetitions for
	flexion /extension

This technique can be performed with a leave-one-out or jackknifing validation method which is often useful for instances where the dataset is small. To ensure that this method could still determine differences between healthy controls and patients of all conditions, all clinical data available was used in this study.

### Dynamic time warping

To ensure that the quantifiable data could be used to distinguish between healthy individuals and all patients (not just patients with MAS 0), all patients who had their data collected were also included in classification tests.

The time series nature of the data allows for various patterns and features to emerge with time along each dimension. We hypothesize that all healthy individuals would present similar patterns or features along each of their dimensions compared to patients. In particular, we believe that the forces exerted by healthy individuals will on average be lower in amplitude compared to clinical data. Furthermore, we believe that the force profiles with respect to position and time that each healthy individual demonstrates will be of a similar pattern or nature and possess similar characteristics. Specifically, one may expect that clinical data will possess higher force magnitudes or spikes in force profiles that characterize a catch or marked increase in force from spasticity.

Grouping each individual based on the magnitude of force as well as these features requires comparisons between each sequence of data collected. Thus, one may expect that two individuals belonging to healthy controls with similar data would be grouped closer together when compared to each other as opposed to data from a patient who experiences a catch. Since the data collected are all continuous values, Euclidean distance is a natural metric. The Mahalanobis distance is also considered which can account for correlations between dimensions [23]. A cosine similarity, a technique that has been studied with EMG signals to detect muscle synergies [24], is also considered as an additional metric.

When comparing two sequences as straight distances or a lock-step measure, time-steps and dimensions are compared on a one to one mapping [25]. The main advantage of the lock-step approach is that it can provide competitive results compared to more sophisticated methods while maintaining a complexity that is still linear to the length of the time series [25]. Dynamic time warping is a widely used algorithm for measuring the similarity between two vectors [26]. It can provide a more intuitive similarity measure, allowing similar patterns to match even if they are out of phase in the time axis. This property may allow for individuals with similar MAS scores to have their force profiles possessing spikes in relatively close proximity to match closer together compared to healthy individuals.

To evaluate the ability to predict MAS scores based on the dataset, an MAS estimation was first made using a nearest neighbour regression. MAS scores of −1 were assigned to healthy individuals so that their scores could be differentiated from MAS 0’s and established as being further away from MAS progressively higher scores greater than 0. The accuracy was then reported as a root mean squared error (RMSE). To perform the MAS approximation, the same distance metrics used in the patient/healthy classification were also used.

Similar to LDA, validation of how well these techniques were at classifying patient/healthy data or approximating MAS was performed with the leave-one-out cross validation procedure. This allowed the entire dataset to be used as a basis of training before comparing the left-out observation to the training set.

Classification was performed with a K-Nearest Neighbour (KNN) classifier since it has been proven to yield comparable if not better accuracy results compared to more complex classification methods [27]. Since a 1NN classifier predicts the label of the nearest neighbour in the set, it reflects directly on the effectiveness of the similarity measure. DTW-based methods have not yet been used to classify spasticity. It is an appropriate procedure for the dataset collected as it can effectively compare temporal sequences of varying lengths. Furthermore, it is capable to comparing inexact pattern matches we would expect to observe in force data as individuals’ with ABI exhibit their abnormal tone through passive motions. Different distance metrics can also be easily substituted to obtain more accurate results. For comparison, a lock-step procedure is also performed as a simple and fast alternative to DTW that can act as a baseline.

## Results

**Fig. 2:**
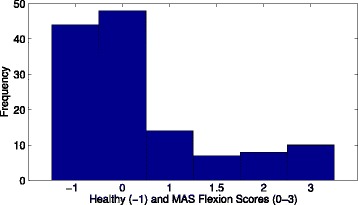
Histograms for MAS scores in study for MAS flexion and MAS extension. MAS Flexion/ Extension (above/below) scores for sample tested in the study. Healthy individuals are represented by the score of −1 while MAS 1+ is coded as 1.5. The majority of those tested had MAS scores of 0

The majority of the 88 limbs of individuals with ABI were found to have scores of MAS 0 or MAS 1, as shown in Fig. 2. The results from healthy individuals were then compared against those collected from individuals with ABI with MAS 0 in either biceps or triceps. The average force exerted by healthy individuals did not differ from patients with MAS 0 (Table 3).

**Table 3:** Welch’s t-test compares the average force of healthy individuals against those with MAS scores of 0. There is no evidence to conclude that average force differs between healthy individuals and patients with MAS 0 scores

Stat	Value
$ \bar {X}_{1} $	9.218
$ \bar {X}_{2} $	9.348
*s*_1_	3.370
*s*_2_	3.355
*s*	0.677
*t*	–0.1909
Deg. of F.	≈93
*P*−*v* *a* *l* *u* *e*	0.849

**Fig. 3:**
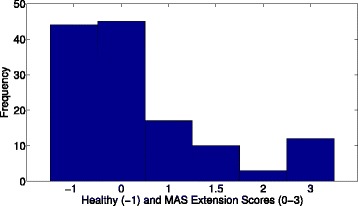
Two axis plot for times series of force and position. Times series plot is shown comparing positions in millimeters on the blue left vertical axis to the forces in Newtons on the right green vertical axis. Force is observed to increase as the time and velocity of each flexion/extension motion increases

A velocity dependent resistance was found for both biceps and triceps (*P*<0.0001) for the robot speeds as shown in Table 4. An example force trace shown in Fig. 3 which shows data for both force and position trace. As time and consequently speed increases, forces are also shown to increase with spikes in the force magnitude readings becoming apparent. Speed level had an effect for the triceps (*P*<0.0001) and approached significance for biceps (*P*=0.054). The interaction between level of health and direction of motion was not found to be significant at the *α*=0.05 level in the tricep model. The effect of each limb had a significant effect in the tricep model (*p*=0.045) but approached significance in the bicep model (*p*=0.066). Terms that had no effect were removed to determine a reduced model with the least squared means shown in Table 5.

**Table 4:** ANOVAs for fixed effects in model 1, comparing healthy individuals to patients for MAS bicep and tricep scores

			MAS Bicep		MAS Tricep	
Fixed Effect	Num df	Den df	F	p	F	p
Health	5	1202	4.55	<0.001	8.75	<0.001
Limb	1	1202	3.39	0.066	4.03	0.045
Speed Level	2	1202	2.92	0.054	7.86	<0.001
Direction	1	1202	28.28	<0.001	22.90	<0.001
Health,Direction Interaction	5	1202	2.84	0.015	1.66	0.142
Robot Speed	12	1202	5.31	<0.001	6.22	<0.001

**Table 5:** Least square mean values of main effects from reduced model where effects that were not found to be significant were removed. Specifically, mean values of forces for Healthy individuals, MAS 0s, extension motions, flexion motions, and speed levels. Means for each effect were found to be significantly different from 0

	MAS Bicep				MAS Tricep			
Effect	Estimate	SE	t	P	Estimate	SE	t	P
Healthy	9.4665	0.5321	17.79	<0.0001	11.2274	0.7548	14.87	<0.0001
MAS 0	10.2071	0.5787	17.64	<0.0001	11.5999	0.7556	15.35	<0.0001
Ext.	10.0529	0.5116	19.65	<0.0001	12.9421	0.8836	14.65	<0.0001
Flx.	12.5678	0.5116	24.56	<0.0001	13.6351	0.7779	17.53	<0.0001
Spd L 1	·	·	·	·	10.9666	0.6336	17.31	<0.0001
Spd L 2	·	·	·	·	11.586	0.5588	20.74	<0.0001
Spd L 3	·	·	·	·	13.4432	0.5588	24.06	<0.0001

The estimated slopes for the velocity dependent resistance is presented in Table 6 for healthy individuals and those with MAS scores of 0. For healthy individuals and those with MAS 0, the slopes corresponding to patient groups were greater in magnitude compared to those from healthy groups. Significant differences exist between each group’s flexion and extension slopes.

**Table 6:** Robot Speed slopes for both healthy individuals and MAS 0’s for both MAS bicep and tricep models. Differences between these slopes for the flexion and extension directions are presented but are not significant

	MAS Bicep		MAS Tricep	
Effect	Healthy	MAS 0	Healthy	MAS 0
Ext.	1.0009	1.7378	0.8006	1.5057
Flx.	0.9593	3.252	1.1595	2.2747
Diff.	–0.0416	1.5142	0.3588	0.769
SE Diff	0.8866	0.8767	0.5914	0.5815

The health-direction interaction was found to have an effect in the MAS tricep model. Thus average forces and slopes in each group should be considered separately. Table 7 presents the least square means for the healthy and MAS 0 groups for both flexion and extension, based on the full models. The differences between flexion/extension motions are also presented and shown to be significantly different. Estimates are presented in Table 8, with a jog speed set to 60 *%* or 0.6. Healthy individuals are compared to all patients and to those with MAS 0. Although Robot Speed was found to have an effect on average force, statistically significant differences were not found for these comparisons.

**Table 7:** Least square mean values of health-direction interaction as well as differences of means from full model

	MAS Bicep		MAS Tricep	
Direction	Healthy	MAS 0	Healthy	MAS 0
Ext.	8.4328	9.1562	8.5691	9.2406
Flx.	10.4383	11.1227	10.575	11.2321
Diff	–2.0055	–1.9665	–2.0059	–1.9915
SE Diff	0.2512	0.2515	0.2499	0.2395

**Table 8:** Estimated differences in means for healthy individuals and patients. Used to assess differences between the two groups at a job speed of 60 %

	MAS Bicep				MAS Tricep			
Contrast	Estimate	SE	t	P	Estimate	SE	t	P
Healthy	9.672	0.816	11.85	<0.001	12.212	0.765	15.97	<0.001
MAS 0	10.188	0.810	12.57	<0.001	12.607	0.765	16.49	<0.001
Patients - Healthy	1.020	1.017	1.00	0.316	1.525	0.951	1.60	0.109
MAS 0 - Healthy	0.516	0.886	0.63	0.530	0.350	0.829	0.42	0.672

The multivariate LDA with all 13 discriminators achieved an accuracy of 61.83 *%*, with leave-one-out validation, for correctly classifying individuals as healthy or as a patient. By gradually reducing the set of discriminators based on those with the lowest weights, classification accuracy was improved to approximately 64.89 *%*. The weight vector obtained from the LDA is summarized in Table 9 for both the first iteration with all discriminators and the final reduced set of 9 with the highest accuracy.

**Table 9:** Vector of weights obtained from multi-dimensional LDA. Discriminators listed are described in Table 2. Results shown in the table are for the first iteration with all discriminators and the final reduced set to obtain the highest accuracy

Discriminate	1st Iteration	Final Iteration
*X*_*maxForce*_	0.0113	0.0065
*Y*_*maxForce*_	–0.0369	–0.0363
*Z*_*maxForce*_	–0.0263	–0.0274
$ \bar {Fx} $	0.0026	0.0028
$ \bar {Fy} $	0.0003	·
$ \bar {Fz} $	0.0012	0.0009
$ \bar {Ftot} $	0.0005	·
*F* *X*_*max*_	0.0000	·
*F* *Y*_*max*_	–0.0003	·
*F* *Z*_*max*_	–0.0003	–0.0002
*F* *t* *o* *t*_*max*_	0.0003	0.0003
$ \hat {F}_{\textit {up}} $	0.0041	0.0038
$ \hat {F}_{\textit {up}} $	0.0013	0.0009

**Fig. 4:**
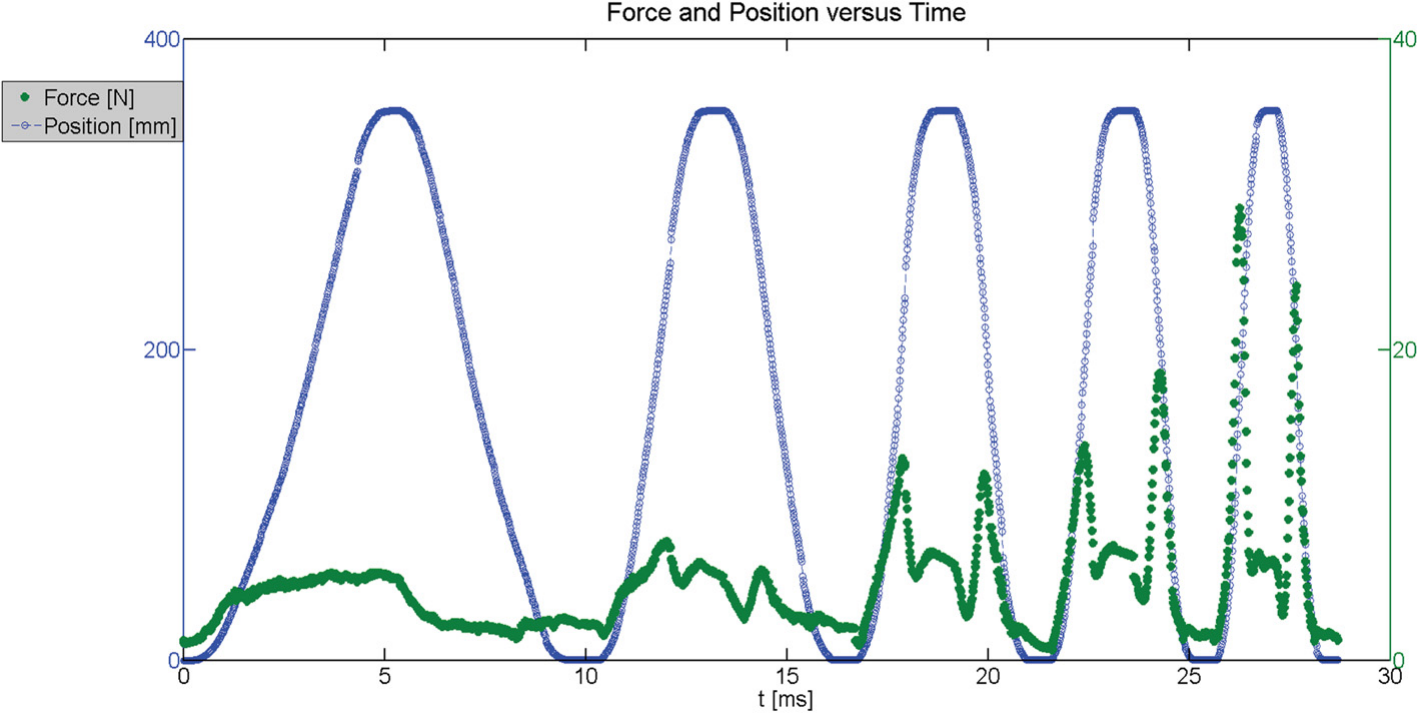
Classification Accuracy with Dynamic Time Warping. Classification accuracy can be improved between patients and healthy individuals by employing the dynamic time warping algorithm. The best accuracy (84.5 *%*) was achieved using the Mahalanobis distance

Results obtained using dynamic time warping were found to improve classification compared to LDA. The results using the Euclidean distance, Mahalanobis distance and cosine distance are presented in Fig. 4. Both Euclidean and Mahalanobis distance were found to exceed accuracy of 80 *%* with maximum values of approximately 84 % and 85.5 *%* respectively using leave-one-out validation.

**Fig. 5:**
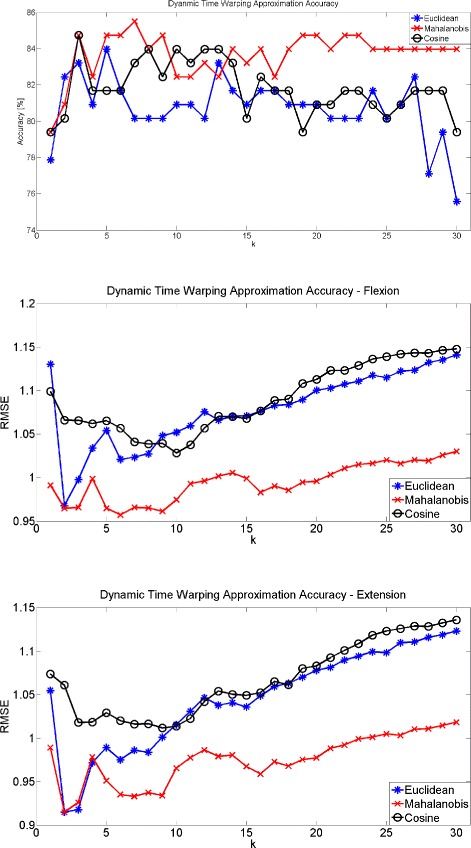
RMSE for MAS flexion and MAS extension estimates. RMSE obtained using DTW with Euclidean Distance, Mahalanobis Distance, and Cosine Similarity. MAS flexion shown above while extension is below

Regression onto to MAS scale and healthy individuals is much more difficult to differentiate amongst the different techniques. The RMSE for both MAS Bicep or Tricep were approximately equal for any number of nearest neighbours (*k*) with the minimum RMSE occurring at either *k*=2 or *k*=6 for Euclidean and Mahalanobis distance. The RMSE for the cosine similarity achieves the lowest error at *k*=9 and *k*=10 for biceps and triceps respectively. The lowest RMSE value achieved is approximately 0.87 for both flexion and extension using the Mahalanobis distance. The results are summarized in Fig. 5.

The maximum leave-one-out accuracy obtained was 85.50 *%* using the Mahalanobis distance with *k*=7. From this trial, those with an MAS score of zero were classified as a patient with an accuracy of 91.07 *%* accuracy.

## Discussion

One important goal of this study was to detect differences between force data obtained from a healthy individual and force data collected from a patient receiving care for spasticity. Clinicians involved with this study identified that patients with ABI and assessed as MAS 0, (indicating no significant spasticity), often report that their limb “does not feel quite right”. This new robot data supports that there may be subtle changes in the muscles resistance that are undetectable with other assessment techniques that explain this phenomenon. The absence of this objective quantitative data to support this patient report makes it challenging for clinicians to help remediate it.

Two key aspects of the data we collected were that it was temporal and multidimensional. Several studies often employ 1-D sensors that report only scalar aggregated representations for patient evaluation, often in the form of a maximum torque or force achieved. For comparison, this study tested scalar representations of resistance to passive motion and results suggested that they were not capable of effectively distinguishing between healthy control data and those from the population of individuals with ABI. Similarly, representing the individual as a set of average force values was unable to accurately differentiate between the two groups. Despite using several statistical tests, it is clear that it is difficult to differentiate between the healthy control group and a group that is primarily individuals with MAS scores of 0. This may suggest that assessment systems for tone that collect 1-D data types such as maximum torques, forces or slopes of either may not be the best approach for the purpose of monitoring. Potential reasons for this include the loss of directional information that occurs when studying the force magnitude as well as the lost of temporal information while averaging.

### Velocity dependent resistance

One promising result is the significant effect of robot velocity on resistance. Healthy individuals were also found to demonstrate velocity dependent resistance. Furthermore, it is also notable that the patient slopes often exceeded those of healthy individuals suggesting the some resistance arising from spasticity was being captured. This suggests the system is capturing relevant information given the most common definition of spasticity from Lance is “velocity-dependent resistance to passive motion” [28]. MAS 0 individuals also had similar slope values, or slightly greater values compared to healthy individuals. This result, however, should not be surprising as those with MAS scores of zero are considered to have no spasticity and would not experience a noticeable velocity dependent resistance. It is possible that had there been a greater number of individuals with MAS scores greater than 0, these observed slopes for patients would be higher as a result of their increased tone. Nevertheless, these findings do, however, provide context to the quantitative results obtained. Assessment systems employing force readings can look for similar results in healthy individuals and while paying special special attention to the extent to which quantitative patient data exceeds that of the control data. This system has the potential to provide clinicians with objective data regarding tone when traditional assessment methods such as the MAS may not have the sensitivity to detect subtle amounts of tone. Exploring the temporal nature of the data and a large set of baseline data allowed for better results for separating the two.

### Dynamic time warping

An important result obtained from the study is demonstrating that the DTW method was most effective at differentiating between healthy baseline data and patient data. The dynamic time warping algorithm is a natural choice as it performs a direct form of comparison between sequences consisting of force and position measurements along three axes. A dynamic time warping of all patients shows that we can achieve >85 *%* accuracy compared to the average force LDA which achieves a maximum of approximately 65 *%*. This is significant as LDA focuses on aspects pertaining to the catch such as the maximum forces achieved and location of those forces. The improved performance by the DTW algorithm suggests that it is more useful to look at the time series as a whole, as opposed to representing an individual’s performance in terms of metrics that summarize different aspects of the passive motion and resistance. For differentiating between healthy individuals and patients, these findings suggest that perhaps it is not the magnitude of force detected from the healthy individuals, but specific features of their forces that are present or absent when compared to those from patients. This new way of analyzing spasticity data may allow for the capturing of any precursory information prior to the catch manifesting, or potential subsequent effects immediately after.

### Importance of positional data

It is notable that position data was found to be important in this study. Although all limb motions were primarily performed in the robot’s y-z plane, it was found that the discriminator *X*_*pos*_, which describes the position along the x-axis where the maximum x-force was read, had to be included or else accuracy would drop to approximately 61 *%*. Referring back to the MAS described in Table 1 we can see that resistance is monitored explicitly along the primary flexion/extension motion. No special attention is paid to resistance not in that plane of motion for the MAS. The results from this study, however, suggest that considering all 3 Cartesian axes is important in distinguishing between patients and healthy individuals.

To fully utilize the data collected, a time series similarity technique was employed to not only consider time and directional information of the forces, but time and directional information of the limb’s position as well. It is interesting to note that in the reduced LDA test, all 3 position-based discriminates were found to be important in maintaining the classification accuracy. This result may suggest that a key aspect in differentiating between healthy and patient data could be position based such as location of the largest magnitude of force. In most healthy individuals, they may be presumed to be at the beginning or end of range the system commences or ends their passive motion. For most patients, however, the maximum force occurs where they could experience a catch as a result of their spasticity as opposed to the beginning or end of range. Such findings are not necessarily attainable using averaging methods such as slopes of forces or torques or EMG signal strength. Furthermore, LDA and DTW both found the 3D positional information to be of importance in obtaining their highest accuracies. This finding provides evidence that 3D positional should be considered when developing new methods to evaluate spasticity.

### Healthy controls and MAS 0

For this study, it is important to recognize that the majority of limbs assessed were scored an MAS of 0. MAS 0 is considered to be an assessment where a catch is not present or detectable. This finding challenges this belief as it is clear that there is a distinguishable difference between healthy baseline values and ABI patients including those with MAS 0 assessments. Therefore, it may be better to compare individuals against a set of quantifiable force data in addition to conventional assessment measures. Although the regression model suggests otherwise, this finding provides strong evidence that the MAS 0 cannot be considered a healthy or fully recovered limb. What an MAS 0 may, instead, represent is another step towards recovery. It also suggests that when treatments are applied to help alleviate spasticity, some resistance can be expected when passively leading the limb for assessment. Therefore quantification of force data may be a necessary step in determining treatment effectiveness of pharmaceuticals, conventional physiotherapy, or occupational therapy. This finding also arms PTs and clinicians with a quantitative result that can assist them in obtaining more rehabilitative therapy, treatments, or necessary resources for individuals under their care who report that their limb doesn’t feel normal. This feeling may be attributable to other positive and negative features of the upper motor neuron syndrome. With the large group of healthy individuals all undergoing similar motions in the study, the system is capable of detecting the subtle differences in these positive and negative features from the patient population to help differentiate them from the healthy group. Future studies may be able to further investigate the roles of these additional features in determining differences between the two groups. Also through further studies, a cut-off or threshold may be determined between healthy and MAS 0 forces. Checking individuals under care against this threshold may lead to cost savings by avoiding unnecessary treatments for those considered healthy, or conversely identifying individuals who still require care. While a force threshold is not currently available, methods such as dynamic time warping technique presented in this paper may be able to aid in this objective.

## Conclusions

This robotic system can collect unique forms of flexion/extension data through the sagittal plane that we can then use to better quantify patient performance. Such quantitative techniques are sought to improve how spasticity in patients is monitored. These methods include logging multi-dimensional time series data, and passively moving the limb through progressively faster motions to gauge velocity-dependent resistance. This quantitative data can provide insight into any changes that may occur in their condition or provide information about the characteristics of the resistance experienced. Results demonstrated that differences exist between individuals of MAS 0 and those who are healthy. This suggests that MAS 0 scores do not reflect perfectly regained ability and perhaps further treatments should be continued if the patient continues to report ongoing issues with their limb. Furthermore, the system was able to demonstrate that considering the force and positional information in 3D provided a more accurate way to perform similarity matching as opposed to using the magnitude of force. Awareness of this information assists clinicians who can now monitor resistance in multiple directions providing a more descriptive representation of a person’s resistance. This information has been demonstrated to be useful and should be considered whereas the conventional method is to manually feel for a non-directional resistance or a system that primarily measures resistance about a single axis.

In future studies, we plan to examine whether or not the robotic assessment can differentiate between all levels of the MAS scores and if the report can be shown to be more sensitive to change than the standard MAS in detecting improvements or deterioration in spasticity. Determining how such quantitative data differs between each level of MAS score may provide clinical insight into how to progress one’s therapy, or which specific indicators to look for while monitoring for changes in spasticity. Awareness of these differences may ultimately lead to a faster determination of treatment effectiveness and a more optimal rehabilitation process for each individual. Often there is pressure to move patients through the health care system very quickly. Medications and various hands-on treatments are used to try to impact spasticity. Sometimes, these treatments and forms of management will include splinting and surgery. Currently it is often several weeks or even months before we can detect a definite change in spasticity as measured by MAS to know if we are on the right track with treatment. The robot’s ability to detect subtle changes in tone would accelerate this process and even increase patient comfort and care. Quantitative methods would provide insight into the individual’s improvement or the effectiveness of specific medications and other treatment options. Alleviating this bottleneck would have a big impact on how quickly decisions related to spasticity treatment could be made which ultimately leads to a quicker recovery and optimization of clinical resources.
